# The Relationship Between College Teachers’ Frustration Tolerance and Academic Performance

**DOI:** 10.3389/fpsyg.2021.564484

**Published:** 2021-03-23

**Authors:** Song Shi, Zizai Zhang, Ying Wang, Huilan Yue, Zede Wang, Songling Qian

**Affiliations:** ^1^School of Education Science, Nantong University, Nantong, China; ^2^Hangzhou Preschool Teachers College, Zhejiang Normal University, Hangzhou, China; ^3^School of Fine Arts, Zhejiang Normal University, Jinhua, China; ^4^School of Teacher Education, Huzhou University, Huzhou, China; ^5^College of Science Education, Jilin Normal University, Siping, China

**Keywords:** college teachers, frustration, academic frustration tolerance, academic performance, theory of constructive failure

## Abstract

The purpose of this study was twofold: to validate the College Teachers’ Academic Frustration Tolerance (CTAFT) Questionnaire and the College Teachers’ Academic Performance (CTAP) Questionnaire and to explore the relationship between frustration tolerance and academic performance among college teachers. A total of 25 experts were recruited to modify and validate both questionnaires, and the results showed that the questionnaires had good content validity. Exploratory factor analysis provided further evidence supporting the reliability of the CTAFT and the CTAP, suggesting that the instruments are reliable and valid. Confirmatory factor analysis showed that frustration tolerance affected academic performance, which could best be modeled in the three dimensions of Affect (AF), Preferred Difficulties (PD), and Action (AC). A total of 450 college teachers from each faculty of both universities were then recruited to explore the significant positive correlation between academic frustration tolerance and academic performance. The results from the structural equation model suggested that AC and PD combined significantly predicted academic performance. To our knowledge, this is the first study to explore the relationship between college teachers’ academic frustration tolerance and academic performance in China.

## Introduction

There are numerous concepts of success and failure. Clifford (1984) used two equations to differentiate between these terms in his performance–goal ratio. Clifford suggested that failure could be defined as having a performance–goal ratio of less than 1 (*F* = *P*/*g* < 1), which means that the goal exceeds its target matching performance. For example, let us say a group of researchers set themselves the goal of publishing their article in the Chinese Social Science Citation Information (CSSCI) journal but are unsuccessful after several attempts. Despite having succeeded in publishing their research article in the Peking University Library Core journal in the process, they still regard themselves as failures, as they failed to achieve their initial goal in submitting their article to a more prestigious journal. On the other hand, success can be objectively defined as having a performance–goal ratio equal to or greater than 1 (*S* = *P*/*g* < 1); people are considered to be successful when they are able to meet and surpass their performance goal.

Frustration is a normative perceptive response to blocked goal attainment, and it often occurs as a consequence of failure (Gelbrich, 2009). Frustration tolerance refers to the ability of individuals to respond to adversity or unmet needs positively and is an indicator of how willing they are to take on challenges (Huang and Lin, 2013). For example, although the hypothetical group of researchers above were unable to achieve their goals, they still maintained a positive mindset, a sign of high frustration tolerance. Academic failure tolerance is a tendency for learners to respond constructively to failure experiences in their academics (Clifford, 1984; Kim and Clifford, 1988). Earlier scientists learned through the theory of helplessness that repeating failures can negatively affect the individuals and emphasized that educational environment helps to avoid failure and frustration (Maier and Seligman, 1976). But then Clifford (1984) presented the theory of constructive failure, proving that in some cases failure can affect positively on an individual and increase their performance. Presented in that context, academic failure tolerance means a tendency to respond in a relatively constructive manner to academic failure outcomes and the results are that students even after a failure will not become helpless and will increase their progress.

Frustration tolerance embodies many characteristics of successful people. Previous research has indicated that frustration tolerance predicts academic achievement and that people with high frustration tolerance have a higher intelligence quotient (IQ) and self-control and are more courageous (Meindl et al., 2019). Additionally, in support of Tan’s (2004) research on adults, which found that irrational beliefs such as low frustration tolerance were associated with stress, Mahon et al. (2007) found a negative relationship between frustration tolerance and individual well-being among young adolescents. Young adolescents with low frustration tolerance were likely to have higher stress levels and a higher chance of suffering from depression and anxiety. Therefore, frustration tolerance is worth further investigation, as it affects people’s lives.

Research on frustration tolerance has focused on students, special needs children, and parents and has been conducted through the use of questionnaires. Most of these studies have explored the different variables associated with student academic achievement and frustration tolerance and have assessed such interpersonal and social factors as self-concept, self-esteem, emotional intelligence, and life adaptation (e.g., Ijaz and Mahmood, 2009; Gulzar et al., 2012). A number of empirical studies have indicated that frustration tolerance has a positive influence on the overcome decreases in grades and other academic pressure (Kim, 2010, 2011). Data from studies also suggest that frustration tolerance is a highly significant correlation with inherent motive (Yoo and Han, 2011). Improving frustration tolerance can help individuals improve their adaptability and satisfaction to the school (Jeon, 2016). Indeed, many researchers have found that frustration tolerance is an internal psychological variable that prevents academic burnout and predicts academic performance (Kim, 1997; Kim and Joo, 1999; Park, 2008; Jo et al., 2013). Frustration tolerance has also been studied in children with special needs. For example, Seymour et al. (2019) compared the differences in frustration tolerance between children with attention-deficit hyperactivity disorder (ADHD) and children without ADHD. The results showed that the children with ADHD quit the Mirror Tracing Persistence Task (MTPT) quicker than children without ADHD, demonstrating a lower level of frustration tolerance. Seymour et al. (2019) concluded that low frustration tolerance is directly linked to ADHD. In addition, findings indicated that frustration intolerance in parents contributed to a high risk of parent–child aggression, which might potentially escalate a child’s risk of maltreatment, from physical discipline to physical abuse (Rodriguez et al., 2017).

Despite the abundance of studies conducted on frustration tolerance, the few instruments available to measure teachers’ frustration tolerance have rarely taken into consideration teachers at the tertiary level of education, not to mention the relationship between college teachers’ frustration tolerance and academic research performance. Zhang (2015) found that rural primary and secondary school teachers had low frustration tolerance, while Liu and Yu (2018) found that the rapid development of higher-education systems put a great deal of pressure on college teachers, especially those with less experience. College teachers for instance have to take on both teaching and academic research responsibilities, and many of them experience a great deal of frustration in their academic research (Wang et al., 2013). However, teachers’ frustration intolerance seems to have been overlooked and therefore needs to be emphasized as people with high frustration tolerance are more positive and constructive when facing difficulties than those who have low frustration tolerance (Clifford et al., 1988).

A large number of instruments for assessing frustration tolerance have been introduced in the field, such as the Survey of Personal Belief (SPB) (Kassinove, 1986) and the Frustration Discomfort Scale (Harrington, 2005), and there is even an instrument specifically for students—the School Failure Tolerance (SFT) scale (Clifford, 1988). Clifford (1988) developed and validated the SFT scale for students in order to identify developmental patterns and sex differences in children’s academic risk-taking and tolerance for failure and confirmed the three anticipated failure tolerance components of Action (AC), Preferred Difficulty (PD), and Affect (AF). The study also revealed the high reliability and validity of SFT for assessing and examining failure tolerance among students. However, there is currently no instrument specifically designed to assess teachers’ frustration tolerance. In order to fill this gap, in the present study a new instrument, the College Teachers’ Academic Frustration Tolerance (CTAFT) Questionnaire, was devised based on Clifford’s (1988) SFT scale, to assess college teachers’ frustration tolerance.

Scientific research is one of the fundamental pillars of academia. Since academic frustration cannot be avoided, a careful analysis of the impact of academic frustration is needed so that its positive effects can be controlled. Also, as there is a positive relationship between frustration tolerance and personal achievement (Rardin and Moan, 1970), it is also necessary to examine the academic performance of teachers. In the present study, an instrument, the Academic Performance Questionnaire, was designed based on Wang (2014), while adding a timeframe in the questions. After examining the reliability and construct validity of both questionnaires, the relationship between college teachers’ frustration tolerance and academic performance could be identified.

Based on the above evidence, the aim of the present research was twofold: first, to develop and validate two instruments, the College Teachers’ Academic Frustration Tolerance (CTAFT) Questionnaire and the College Teachers’ Academic Performance (CTAF) Questionnaire, which assess college teachers’ frustration tolerance and their academic performance, respectively, and second, to determine the relationship between college teachers’ frustration tolerance and their academic performance.

## Materials and Methods

The research consisted of two parts: the first part was concerned with the modification and validation checking of the questionnaires, the second part with finding the relationship between frustration tolerance and academic achievement through the questionnaires.

### Participants and Procedure

#### Participants and Procedure for Questionnaire Modification

The step for questionnaire modification and valid and reliable construction is based on the procedure provided by Churchill (1979) and Hinkin et al. (1997). The drafts of both questionnaires were prepared based on the existing literature. Twelve college teachers (eight of whom had a doctorate degree) were invited to make modifications to the contents of the questionnaires and included deleting, adding, or assessing the content. After this, another 13 college teachers, from different universities, were asked to suggest final modifications and invited to evaluate the content validity of the two questionnaires—final questionnaire generation. Among these experts, one had obtained a master’s degree and twelve had obtained a doctoral degree. The experts included three professors, nine associate professors, and one lecturer. The Item-Content Validity Index (I-CVI) was used to calculate the content validity of the questionnaires quantitatively.

#### Participants and Procedure for Determining the Relationship Between Frustration Tolerance and Academic Achievement

A total of 450 participants were recruited from a National Key University in Northeast China and a normal university in Zhejiang Province, respectively. The universities are located in the Northern and Southern parts of China to represent different cultural contexts, but both of them are in Eastern China, where economics is relatively developed. Teachers from the same university were assigned to the same group sampling unit. Both colleges cover subjects in arts, science, and engineering, including Marxism, educational, mathematics, physics, and fine arts departments. Online questionnaires were distributed to each of the faculties of both universities. A total of 450 questionnaires were received. Thirty-three participants who took less than 60 s to fill in the questionnaire were excluded from the analysis, leaving 417 valid samples, with 173 male teachers (41.5%) and 244 female teachers (58.5%). Among the participants, there were 23 teaching assistants (5.5%), 180 lecturers (43.2%), 152 associate professors (36.5%), and 62 professors (14.9%). Two hundred and forty-two participants had obtained a doctorate degree (58.0%), 175 had a masters or bachelor’s degree (42.0%), 41 had an overseas degree (9.8%), and 145 were postgraduate supervisors (34.8%).

### Measures

#### Item-Content Validity Index (I-CVI)

The I-CVI (Rodrigues et al., 2017) was used to calculate the content validity of the questionnaires quantitatively. The I-CVI is computed as the number of experts giving a rating of “very relevant” for each item divided by the total number of experts. It is administered via questionnaires using a 4-point Likert scale: irrelevant, weakly relevant, relevant, and strongly relevant. The values range from 0 to 1, where if I-CVI > 0.79, the item is relevant; if between 0.70 and 0.79, it needs revisions; and if below 0.7, the item is eliminated (Zamanzadeh et al., 2015).

#### College Teachers’ Academic Frustration Tolerance Questionnaire (CTAFT)

The CTAFT was adapted from Clifford’s (1988) School Failure Tolerance (SFT) scale specifically to assess college teachers’ academic performance and included 22 items. The questionnaire consisted of three subscales covering three dimensions: Affect, with 9 items, three of which were excluded after the CVI check, leaving 6 items mainly concerning the negative emotional responses of college teachers when encountering academic difficulties; Preferred Difficulty, consisting of 5 items mainly concerning college teachers’ willingness to engage in challenging research; and Action, consisting of 8 items, one of which was excluded after the CVI check, leaving 7 subitems mainly concerning college teachers’ actions when facing academic difficulties. At the same time, some of the original items were not included in the modified version of the questionnaire, such as “I feel terrible if I made a wrong answer.” However, some specific scenarios related to college teachers’ academic behaviors were included in the questionnaire, such as “I worked hard in my research, but the experience of frustration weakened my enthusiasm or interest in academic research.” The final version of the CTAFT questionnaire thus contained a total of 18 items.

The CTAFT was administered via a questionnaire using a 5-point Likert scale, with 1 = very inconsistent; 2 = relatively inconsistent; 3 = general; 4 = relatively consistent; and 5 = very consistent. After the questionnaires were collected, the scoring method was then reversed such that the higher the total score of the particular dimension, the stronger the college teachers’ academic frustration tolerance. Higher scores on subscales or in general represented individuals with high frustration tolerance. Twenty-seven percent above and below the total mean score was classified as the upper- and lower-bound groups, respectively. For the frustration tolerance questionnaire, scores of 64 points or above represented the high score group, while 52 points or below represented the low score group.

#### College Teachers’ Academic Performance Questionnaire (CTAP)

The CTAP was revised and modified from Wang’s (2014) Academic Performance Questionnaire and was administered online. The questionnaire included 7 items; however, after a content validity check, 2 items were excluded, leaving a total of 5 items in the questionnaire. The questionnaires used a 5-point Likert scale, with 1 = very inconsistent; 2 = relatively inconsistent; 3 = general; 4 = relatively consistent; and 5 = very consistent. Higher scores represented higher academic achievement. Participants scoring 16 points or above were classified as the high-scoring group, while those scoring 10 points or below were classified as the low-scoring group.

## Results

### Content Validity of the Two Questionnaires

The I-CVI was used by 13 experts to measure the content validity of the two questionnaires. If the I-CVI value of the item was lower than 0.7, the item would be excluded (see Table 1). Items in the College Teachers’ Academic Frustration Tolerance (CTAFT) Questionnaire with an I-CVI value lower than 0.7 included “I am afraid to be seen as weak in academia,” with a value of 0.69; “I feel unfairly treated when I know someone who had obtained academic achievements through unethical behaviors,” with a value of 0.54; and “I like to ask ‘why’ when working on academic research,” with a value of 0.62.

**TABLE 1:** Code and contents in each dimension and the I-CVI value of CTAFT and CTAP.

College Teacher’s Academic Frustration Tolerance Questionnaire (CTAFT)			
**Dimension**	**Code**	**Contents**	**I-CVI**
Affect (AF)	A1	I felt sad about my poor academic performance over the past 3 years.	0.85
	A2	I’m afraid of making mistakes when doing academic research.	0.77
	A3	I encountered difficulties in conducting research, such as rejection of publishing a paper, I felt frustrated and became emotional.	0.92
	A4	I feel frustrated when I see others earn more and achieve more than me.	0.77
	A5	The frustration that I have experienced in academia, diminished my enthusiasm or interest in doing research.	0.85
	A6	I worry about not being able to finish my project on time.	0.85
Preferred difficulty (PD)	B1	I love research that is challenging.	0.92
	B2	I prefer engaging in face-paced than time-consuming research.	0.92
	B3	In order to have higher-level of academic achievements, I am willing to “sharpen one sword in 10 years.”	0.85
	B4	I am willing to learn the latest research methodology.	0.85
	B5	I set myself high standards when conducting research.	0.77
Action (AC)	C1	I tend to give up when my research is not going well.	0.77
	C2	I am willing to conduct a valuable new research, even if I don’t have much previous experience on it.	0.85
	C3	I will try my best to solve the problems existing in the research, such as limited time, insufficient funds, poor working atmosphere, unsupportive team, etc.	1
	C4	I learn from mistakes.	1
	C5	I will work hard in the academic field.	1
	C6	I will learn from those who are better than me.	0.85
	C7	In order to achieve my goals in making successful project application, I will be well prepared.	0.77

**College Teachers’ Academic Performance Questionnaire (CTAP)**			

**Dimension**	**Code**	**Contents**	**I-CVI**

Academic performance	D1	I have published papers on scholarly journals over the past 3 years.	1
	D2	I published more research paper than other colleagues in the department over the past 3 years.	0.92
	D3	The number of scientific research that I have completed over the past 5 years is above the department average.	1
	D4	I have won more scientific research awards than most of my colleagues in the Department over the past 3 years.	0.92
	D5	I have been above the department average in other academic achievements.	0.85

Items in the College Teacher’s Academic Performance (CTAP) Questionnaire with an I-CVI value lower than 0.7 were excluded and included “I have published reports or have been an academic conference speaker over the last 3 years,” with a value of 0.62 and “I have helped others in their research work over the last 3 years,” with a value of 0.54.

The Scale-Content Validity Index/Universal Agreement (S-CVI/UA) is used to determine the validity of the questionnaire. It is calculated by adding all items with I-CVI equal to 1 divided by the total number of items (Haron et al., 2019). In order to have good content validity, the S-CVI/UA should be equal to or more than 0.8 (Shi et al., 2012). Overall, with the use of the Universal Agreement method in understanding the relevancy of the overall questionnaire, the CTAFT demonstrated poor content validity. Only three items were recognized as “strongly relevant” and “relevant” by the experts. For the CTAP, the S-CVI/UA demonstrated low content validity, with only two items being selected as “strongly relevant” and “relevant” and no items selected as “irrelevant.” Notably, although the S-CVI/UA value did not suggest good content validity, this might have been due to the large sample size and because research on college teachers’ frustration tolerance and academic performance is a newly developed topic that has not been studied previously in China. Therefore, in order to achieve acceptable content validity, only items with I-CVI higher than 0.7 would be included in the questionnaire.

### Main Analysis of the CTAFT and the CTAP

The average mark of the CTAFT was between 2.58 and 3.78, and the standard deviation was between 0.90 and 1.25. The average number of academic performance questionnaires was between 2.35 and 2.86, and the standard deviation was between 1.14 and 1.19. The total scores of the frustration tolerance questionnaire and the academic performance questionnaire were calculated by comparing extreme groups. The independent sample *t*-test showed that all items in both questionnaires were significant.

Confirmatory factor analysis was conducted to examine whether the three dimensions, AF, PD, and AC in CTAFT, measured a single latent construct. As items with factor loadings lower than 0.5 were excluded, items B2 and C1, with factor loadings of 0.18 and 0.13, respectively, were not included in the analysis. Hence, the lowest factor loading of confirmatory factor analysis was C2 (0.53), with the others all above 0.6. The error variances were all positive (0.26–0.75). The C.R. values of all error variances were between 5.37 and 13.50, which are significant at the level of 0.001. The standard errors of parameters were between 0.024 and 0.145.

Several widely used model fit indexes were used to evaluate the model fit, including the chi-square value (χ^2^); the comparative fit index (CFI); the Tucker–Lewis index (TLI); the root mean square error of approximation (RMSEA); the standardized root mean square residual (SRMR); and the weighted root mean square residual (WRMR). The overall fit of the model was good, with results showing χ^2^ = 333.857, *p* < 0.001, df = 101, GFI = 0.904, AGFI = 0.871, NFI = 0.897, CFI = 0.925, RMR = 0.056, RMSEA = 0.074. However, some indicators were not significant. After items C2 and C4 items were excluded, χ^2^ = 192.514, *p* < 0.001, df = 74; absolute fit indices GFI = 0.937 > 0.9, AGFI = 0.911 > 0.9, RMSEA = 0.062 < 0.008; incremental fit indices NFI = 0.932 > 0.9, RFI = 0.916 > 0.9, IFI = 0.957 > 0.9, MNFI = 0.947 > 0.9, CFI = 0.957 > 0.9, PGFI = 0.661 > 0.5, PNFI = 0.758 > 0.5, CN = 206 > 200, NC = 2.602 < 3, AIC = 254.514 CAIC = 410.540. The absolute moderate index, incremental fit indices, and the simple moderate index were statistically significant, indicating a good model fit and high internal consistency reliability (Kline, 2005; Hair et al., 2006).

### Reliability of the CTAFT

In addition, in terms of individual reliability, the individual reliability of 14 observation indicators was 0.34, which is lower than 0.4. However, except for C3, the values of all the indicators were between 0.41 and 0.70. The composite reliability values of AF, PD, and AC were 0.88, 0.83, and 0.85, respectively. All the composite reliability values of the three potential scalars were greater than 0.080. In terms of average variation extraction, AF, PD, and AC values were 0.56, 0.55, and 0.58, respectively, all of which are greater than 0.05. In terms of use, the nested confirmatory factor analysis model was used to investigate the discriminant validity (Hatcher, 1994; Ahire et al., 1996), and the model competition method was used to compare the χ^2^ difference between the unrestricted and restricted models. The χ^2^ difference between the two models of the three dimensions was greater than 3.84, reaching a significant difference level, which indicated good discrimination between dimensions (Anderson, 1987; Anderson and Gerbing, 1988; Venkatraman, 1989), as shown in Table 2.

**TABLE 2:** A summary of dimension discrimination competition model.

Groups	Restricted model	Unrestricted model	Δχ^2^
AF and PD	261.14	74.36	186.78
AF and AC	322.30	90.32	231.98
PD and AC	218.55	76.23	142.32

**FIGURE 1:**
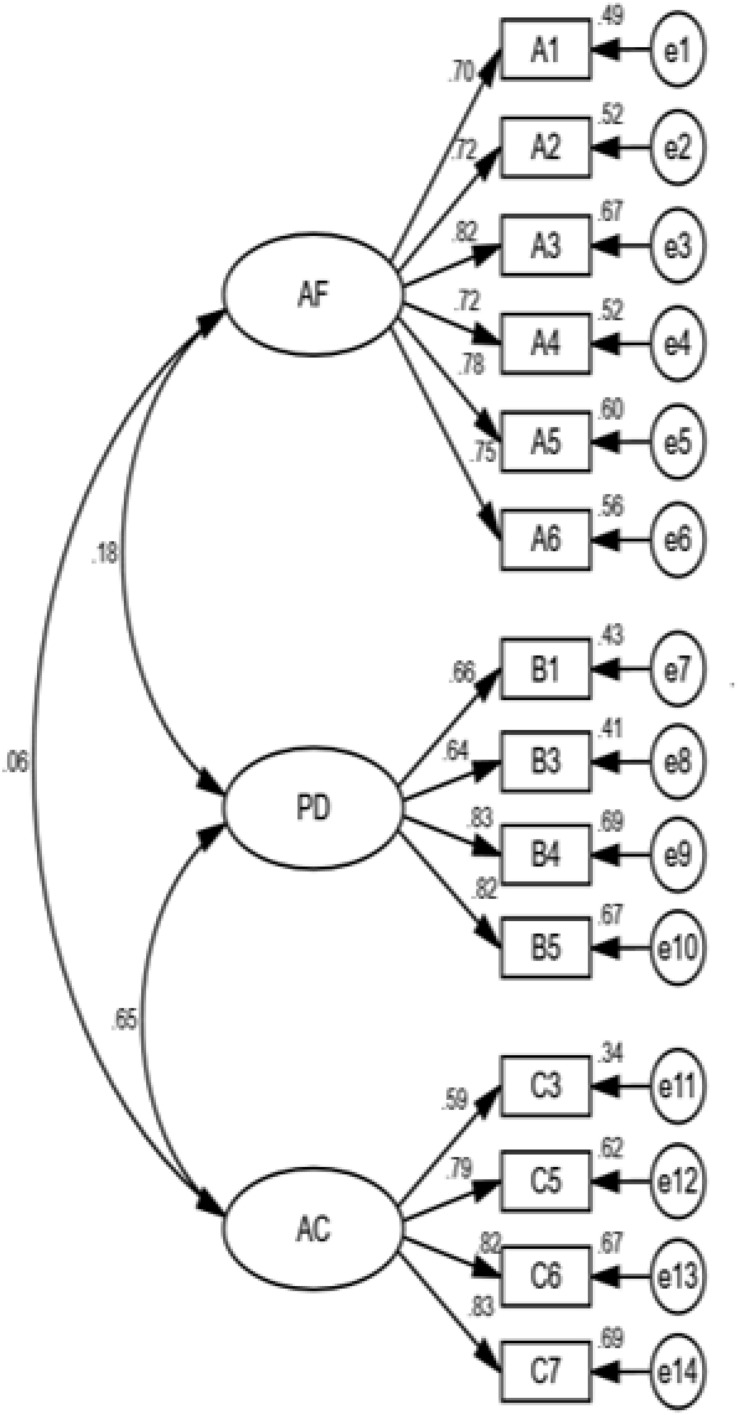
The 3-dimensional confirmatory factor model (left).

To sum up, the CTAFT included 6 items in AF, and PD and AC each included 4 items. All indicators were significant, suggesting a good model fit with high internal consistency reliability and individual reliability, as shown in Figure 1. The results of the exploratory factor analysis were consistent, with confirmatory factor analysis showing that the three dimensions could explain 64.34% of the variation in academic frustration tolerance.

The correlation between AC and AF and the correlation between PD and AF were not statistically significant, but the correlation between AC and PD was statistically significant. While Zhang and Yang (1969) defined frustration tolerance as the ability of an individual to withstand challenges and avoid behavioral disorders, Zhou (2003) pointed out that frustration tolerance includes the ability to avoid psychological disorders. Considering the findings and the significant correlation between AC and PD in the current research, AC and PD were combined into a new dimension, “Preferred Action” (PA), in the following confirmatory factor analysis.

**FIGURE 2:**
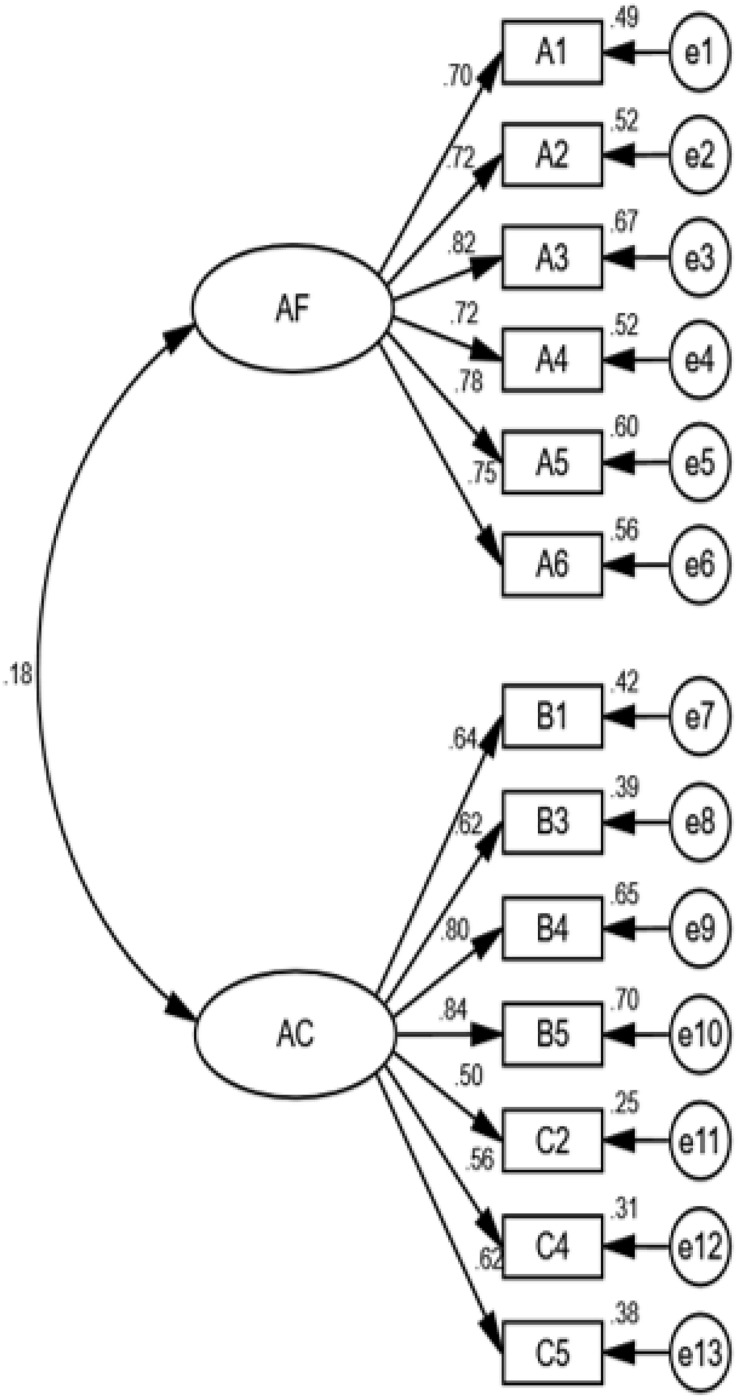
The 2-dimensional confirmatory factor model.

Confirmatory factor analysis was conducted, and items B2 and C1, with factor loadings less than 0.05, were excluded in the analysis. Item B3 (0.52) had the lowest factor loading, 5 items had factor loadings below 0.06, and the rest of the items had factor loadings above 0.07. The error variances were all positive with factors ranging from 0.95 to 1.34. The C.R. values of all error variances ranged between 9.16 and 14.76, which were significant at the level of 0.0001. There were no significant standard errors on items with factor loads between 0.0074 and 0.0113, which indicated a good model fit. However, with reference to the M.I. value of the revised model, the overall model was not significant. After excluding items C6, C3, and C7, χ^2^ = 162.313, *p* < 0.001, df = 64, absolute fit indices GFI = 0.943 > 0.9, AGFI = 0.918 > 0.9, RMSEA = 0.061 < 0.08, incremental fit indices NFI = 0.930 > 0.9, RFI = 0.914 > 0.9, IFI = 0.956 > 0.9, NNFI = 0.946 > 0.9 CFI = 0.956 > 0.9, PGFI = 0.663 > 0.5, PNFI = 0.763 > 0.5, CN = 215.0 > 200, NC = 2.536 < 3, AIC = 216.313, CAIC = 352.206. Absolute fit indices, incremental fit indices, and parsimonious fit indices were all significant (Kline, 2005; Hair et al., 2006), with desirable model fit, as shown in Figure 2. A comparison of the dimensions showed that PA retained 4 items from PD, and only one item (C5) from AC. Items C3, C6, and C7, which reflected academic actions, were excluded in the 2-dimensional confirmatory factor analysis. Although the 2-dimensional and 1-intermediate confirmatory factor models were adequate and had an acceptable fit, the 3-dimensional model was still a fundamentally better fit.

### Validity and Reliability of the CTAP

Principal component analysis was used to examine the reliability of the CTAP questionnaire. Only items whose eigenvalues were greater than 1 were included in the analysis. The KMO value was 0.0859, χ^2^ = 1465.32, df = 10, *p* < 0.001. The characteristic values of the 5 items were higher than 0.5. Only 1 item had an eigenvalue greater than 1 after the sum of loading square, *SD* = 72.42%, and the component load of each item was greater than 0.7, Cronbach’s alpha = 0.902, as shown in Table 3.

**TABLE 3:** Summary of factor analysis on academic performance.

Items	Average	Standard deviation	Factor loadings	Commonality	Corrected item and total correlation	Variance explained
D1	0.86	1.19	0.72	0.52	0.60	72.42%
D2	2.49	1.18	0.93	0.86	0.87	
D3	2.45	1.18	0.91	0.83	0.84	
D4	2.35	1.14	0.86	0.74	0.77	
D5	2.55	1.15	0.82	0.68	0.72	

### Relationship Between Academic Frustration Tolerance and Academic Performance

There was a positive correlation between the three dimensions of academic frustration tolerance and academic performance, with *r* = 0.43, *p* < 0.001 (see Table 4). Based on maximum likelihood estimation, the standardized regression coefficient between academic frustration tolerance and academic performance was 0.43 *p* = 0.005 < 0.01, with *X*^2^ = 90.532, *p* < 0.01, df = 19, absolute fitness index GFI = 0.950 > 0.9; AGFI = 0.905 > 0.9; RMSEA = 0.095 > 0.08; value-added fitness index NFI = 0.9 49 > 0.9, RFI = 0.924 > 0.9; IFI = 0.959 > 0.9; NNFI = 0.939 > 0.9, CFI = 0.959 > 0.9; PGFI = 0.501 > 0.5; PNFI = 0.644 > 0.5; and CN = 139.00 > 200. The RMSEA index was not statistically significant.

**TABLE 4:** Descriptive statistics of the study variables.

Variables	Mean	*SD*	AF	PD	AC
AF	16.97	5.70			
PD	13.26	3.26	0.149**		
AC	14.50	3.08	0.040	0.565***	
Academic performance	12.69	4.95	0.278***	0.311***	0.329***

**p < 0.01, ***p < 0.001.

Based on the maximum likelihood method, the standardized regression coefficient between academic frustration tolerance and academic performance was 0.43, *p* = 0.005 < 0.01. *X*^2^ = 90.532, *p* < 0.001, df = 19; absolute fitness index GFI = 0.950 > 0.9; AGFI = 0.905 > 0.9; RMSEA = 0.095 > 0.08; value-added fitness index NFI = 0.9 49 > 0.9; RFI = 0.924 > 0.9; IFI = 0.959 > 0.9; NNFI = 0.939 > 0.9; CFI = 0.959 > 0.9, PGFI = 0.501 > 0.5; PNFI = 0.644 > 0.5; and CN = 139.00 > 200. RMSEA was not desirable for an acceptable model, as the value was not smaller than0.08.

According to the model modification of M.I., E4 and E5 had relatively large MI values, which meant that there might be an overlap between item D4 (“I have won more scientific research awards than most of my colleagues in the Department over the past 3 years.”) and item D5 (“In terms of academic achievement, I am slightly above average.”). Factor loadings of potential variables were not significant as a measurement index of academic frustration tolerance, with a value = 0.13 < 0.5.

**FIGURE 3:**
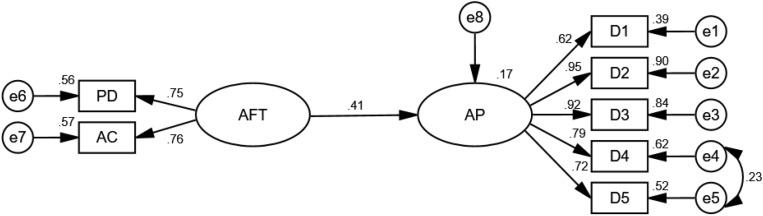
A model of the relationship between academic frustration tolerance and academic performance.

The revised model showed that the 6 regression weighted values were significant, *p* < 0.0001 (Figure 3), where 2 reference indexes—academic performance to D1 and academic frustration tolerance to PD—were excluded. The variances of the 9 exogenous variables were significant with *p* < 0.001. The standard errors of the parameters were between 0.02 and 0.91, with good internal quality, χ^2^ = 37.03, df = 12, *p* < 0.001. In other indicators, GFI = 0.974 > 0.9, AGFI = 0.940 > 0.9, RMSEA = 0.071 < 0.08, incremental fit index NFI = 0.978 > 0.9, RFI = 0.962 > 0.9, IFI = 0.985 > 9, NNFI = 0.974 > 0.9, CFI = 0.985 > 0.9, parsimonious fit index PNFI = 0.559 > 0.5, AIC = 69.029, CAIC = 149.559, CN = 237.00 > 200. The total effect of academic frustration tolerance on academic performance was 0.41, which could explain 17% variation in academic performance.

### Moderators of the Relationship Between Academic Frustration Tolerance and Academic Performance

Sex had no moderating effect on the relationship between academic frustration tolerance and academic performance (*p* = 0.151 > 0.05, Cmin = 2.059). The location in which the teachers obtained their degrees from had no moderating effect on the relationship between R&D frustration tolerance and R&D performance (*p* = 0.628 > 0.05, CMIN = 0.234). Educational level had no moderating effect on the relationship between academic frustration tolerance and academic performance (*p* = 0.114 > 0.05, Cmin = 2.495). The two hypothetical models were divided into low professional title (teaching assistant and lecturer) and high professional title (Associate Professor and Professor) for multigroup analysis, with path coefficients of 0.10 and 0.18, respectively.

**FIGURE 4:**
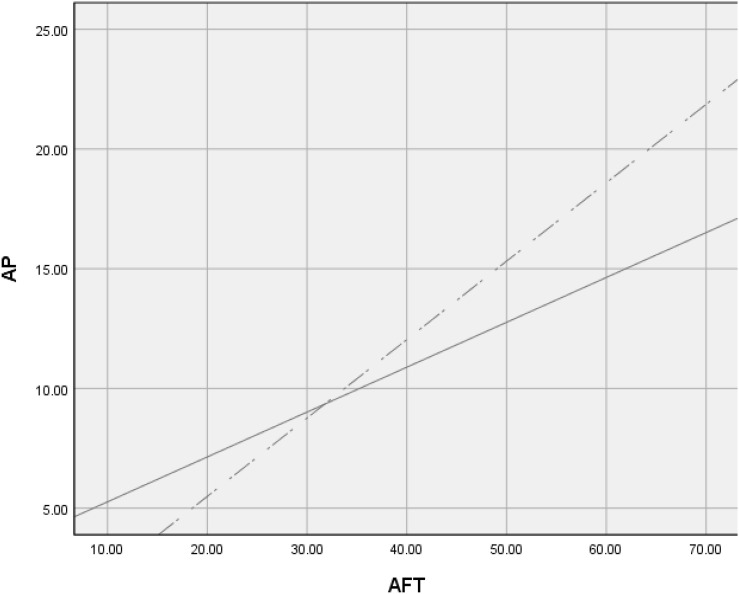
Professional title as moderator. The dotted line represents participants high professional title (*R*^2^ = 0.0250), and the solid line represents participants low professional title (*R*^2^ = 0.0142).

The restricted model and the unrestricted model of Δχ^2^ = 5.642 were significant (df = 1, *p* = 0.018 < 0.05), while ΔNFI = 0.0036, ΔIFI = 0.003, ΔRFI = 0.002, ΔTLI = 0.002; all met the criteria of having the value less than 0.05. The correlation between academic frustration tolerance and academic performance was regulated by the level of professional title. As shown in Figure 4, a high professional title moderated the relationship between academic frustration tolerance and academic performance.

**FIGURE 5:**
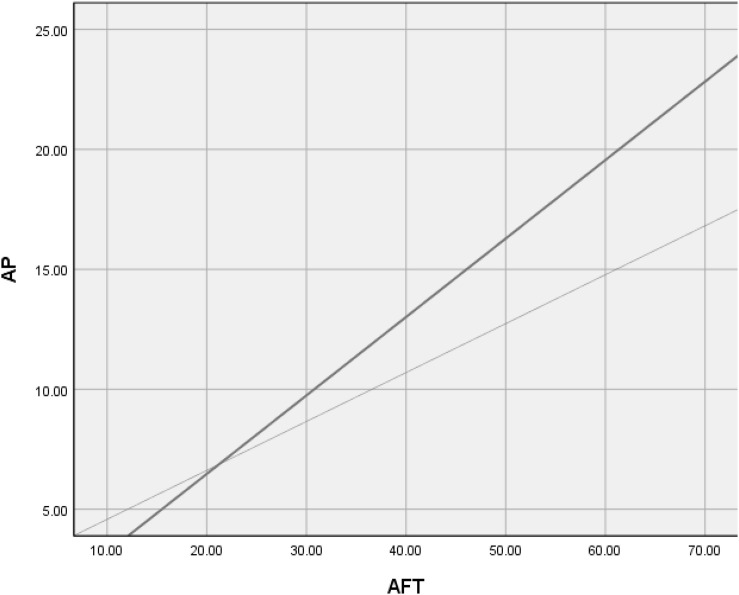
Master supervisors as moderator. The thin line represents those who are not master supervisor (*R*^2^ = 0.149), and the thick line represents the master supervisors (*R*^2^ = 0.276).

The Δχ^2^ = 10.626 of the restricted model and the unrestricted model were significant (df = 1, *p* = 0.001 < 0.05), while ΔNFI = 0.006, ΔIFI = 0.007, ΔRFI = 0.005, and ΔTLI = 0.005 all met the criteria of having the value less than 0.05, as shown in Figure 5.

## Discussion

The findings showed that academic performance improved along with increasing academic frustration tolerance and that PD and AC frustration tolerance had a major influence on academic performance. Frustration tolerance includes the emotional response of individuals after experiencing failure, developing plans and programs to recover from failure, actions to overcome failure, facing possible failure, and likeness to challenge difficult tasks or not, etc. (Kim, 2002). From the concept of frustration tolerance, it can be seen that individuals are not frustrated by the failure and can challenge emotion, cognition, and behavior again. According to the theory of constructive failure (Clifford, 1984), it was found that failure sometimes stimulates positive and constructive responses in particular conditions. In other words, in the face of setbacks, some people are able to overcome negative circumstances, learn from the experience, constantly pursue their goals, and successfully complete tasks, while others cannot (Lee, 2018).

In order to improve academic performance and have better academic achievements, the following is suggested. First, academic goals should be achievable, allowing them to be within college teachers’ zone of proximal development. College teachers should have clear academic goals and divide their goals into actionable steps. For example, to become an associate professor, a teacher should know the evaluation requirements of the university and stay self-motivated in order to become a more accomplished academic. In addition, academic achievements can have a motivational effect on teaching performance. Even if teachers experience academic frustration, they should be humble and find ways to improve, rather than look for excuses, such as blaming their college when their journals were not published. Furthermore, teachers should look at failure as an opportunity for reflection, by asking themselves why a particular experiment or project application failed. By taking these factors into consideration, people are able to achieve higher standards in their academic performance.

There are many factors contributing to one’s frustration tolerance, such as family and personal experience; personal background, psychological factors, physical abilities, religion, social environment, and interpersonal relationships (Hybl and Stagner, 1952; Wu et al., 2015). Hence, individuals experience frustration differently. Harrington (2011) suggested a cognitive behavioral therapy as a means of improving an individual’s frustration intolerance, for example through “Rational Emotional Behavior Therapy” (REBT)—this therapy aims to change an individual’s response to life’s hardship to healthy reactions, by reminding the individual that frustration and discomfort are feelings that everyone experiences in their lives. There is a common misunderstanding that this therapy might help people become more effective in avoiding failure or controlling feelings of discomfort and frustration. However, in considering using REBT to help individuals with frustration intolerance, the most important thing to keep in mind is to distinguish frustration intolerance beliefs from those of self-worth and to maintain a healthy and rational mind to help in achieving our personal goals.

According to the existing research, the more frustration an individual experiences, the lower frustration tolerance is (Lee, 2003). For example, if the university teachers’ frustration about their research is generally low, this may be related to the researching environment in the university (Li et al., 2018). In that case, the higher scientific research performance appraisal system will bring strong negative effects (Baron and Kenny, 1986; Bao and Wang, 2012; Wang et al., 2014). It will increase the frustration experience of teachers in scientific research, and the increase of frustration experience will reduce the frustration tolerance. In addition, the response and support of universities to teachers’ scientific research will also affect teachers’ scientific research tolerance (Quan, 2019). However, most university administrators ignore the process of scientific research and only focus on results, by which the universities can obtain national-level topics and publish high-level articles (Cui, 2018; Liu and Yu, 2018). Therefore, universities should set up appropriate academic assessment goals and provide academic research support to teachers. With the increasing of school education experience, academic frustration tolerance will decrease (Kim, 1994). There are also two topics that need further researches: how will the frustration tolerance increase with teachers’ age and what are the relevant influencing variables, but due to the limitations of the sample in this study, these research goals cannot be completed. Further studies should examine other factors that might directly affect university teachers’ academic frustration tolerance, especially with the regard to the overall academic system and research development. Moreover, the relationships between external environment and academic frustration tolerance should be explored further in order to take specific countermeasures for improvement.

## Conclusion

In the present research, we developed and validated two questionnaires: The College Teachers’ Academic Frustration Tolerance (CTAFT) Questionnaire and the College Teachers’ Academic Performance (CTAP) Questionnaire. Both questionnaires were found to have good reliability and validity. Through confirmatory factor analysis, the CTAFT questionnaire was found to contain three dimensions: AF, PD, and AC. Absolute fit indices, incremental fit measurement, and parsimonious fit indices were all significant, with good model fit. There was a significant positive correlation between academic frustration tolerance and academic performance. In the structural equation model composed of PD and AC, academic frustration tolerance had significant predictive power with respect to academic performance.

## Data Availability Statement

The raw data supporting the conclusions of this article will be made available by the authors, without undue reservation.

## Ethics Statement

The studies involving human participants were reviewed and approved by Ethic Committee of Jilin Normal University. The patients/participants provided their written informed consent to participate in this study.

## Author Contributions

SS and HY designed the study and wrote the protocol. ZW and SQ carried out the data analysis and data collection, and drafted the manuscript. ZZ and YW conducted the critical revision. All authors contributed to the article and approved the submitted version.

## Conflict of Interest

The authors declare that the research was conducted in the absence of any commercial or financial relationships that could be construed as a potential conflict of interest.
