# Corrigendum: The Relationship Between College Teachers' Frustration Tolerance and Academic Performance

**DOI:** 10.3389/fpsyg.2021.687055

**Published:** 2021-05-05

**Authors:** Song Shi, Zizai Zhang, Ying Wang, Huilan Yue, Zede Wang, Songling Qian

**Affiliations:** ^1^School of Education Science, Nantong University, Nantong, China; ^2^Hangzhou Preschool Teachers College, Zhejiang Normal University, Hangzhou, China; ^3^School of Fine Arts, Zhejiang Normal University, Jinhua, China; ^4^School of Teacher Education, Huzhou University, Huzhou, China; ^5^College of Science Education, Jilin Normal University, Siping, China

**Keywords:** college teachers, frustration, academic frustration tolerance, academic performance, theory of constructive failure

In the published article, there was an error in affiliation 2. Instead of “Faculty of Education,” it should be “Hangzhou Preschool Teachers College.” There was also an error in affiliation 3. Instead of “Hangzhou,” it should be “Jinhua.”

The authors apologize for these errors and state that they do not change the scientific conclusions of the article in any way. The original article has been updated.

